# A compilation of North American tree provenance trials and relevant historical climate data for seven species

**DOI:** 10.1038/s41597-021-00820-2

**Published:** 2021-01-26

**Authors:** Clara Risk, Daniel W. McKenney, John Pedlar, Pengxin Lu

**Affiliations:** 1 Ontario Forest Research Institute, Ontario Ministry of Natural Resources and Forestry, 1235 Queen Street East, Sault Ste Marie, ON P6A 2E5 Canada; 2 Great Lakes Forestry Centre, Canadian Forest Service, Natural Resources Canada, 1219 Queen Street East, Sault Ste Marie, ON P6A 2E5 Canada

**Keywords:** Forestry, Forestry

## Abstract

Tree provenance trials consist of a variety of seed sources (or provenances) planted at several test sites across the range of a species. The resulting plantations are typically measured periodically to investigate provenance performance in relation to abiotic conditions, particularly climate. These trials are expensive and time consuming to establish, but are an important resource for seed transfer systems, which aim to match planting sites with well-adapted (climatically suitable) seed sources. Provenance trial measurements may be underutilized because the data are scattered across publications, conference proceedings, and university theses. Here we document an effort to collect available provenance trial measurements and associated climate data for seven eastern North American tree species (*Pinus strobus*, *Pinus banksiana*, *Picea glauca, Picea mariana, Quercus rubra, Larix laricina, Betula alleghaniensis*). The resulting datasets included a total of 773 provenances and 62 test sites, with 65 historical climate variables appended to each location. We hope this data will support forest managers in making seed transfer decisions, particularly in an era of rapid climate change.

## Abstract (summary)

**Table:** 

Measurement(s)	height • survival • phenology
Technology Type(s)	digital curation
Sample Characteristic - Organism	Pinus strobus • Pinus banksiana • Picea glauca • Picea mariana • Quercus rubra • Larix laricina • Betula alleghaniensis
Sample Characteristic - Environment	woodland area
Sample Characteristic - Location	Canada • United States of America

Machine-accessible metadata file describing the reported data: 10.6084/m9.figshare.13136060

## Background & Summary

Natural populations of tree species often vary in traits such as cold hardiness, growth, and bud phenology across their geographic range, due to local adaptation^1^. Much research has been done to quantify the variation in these traits across a species’ range for the purpose of elucidating population-level preferences in environmental conditions. Generally, tree provenance studies involve collecting representative seed of natural populations from a variety of locations (also referred to as provenances) across the range of a species and subsequently planting them at one or more test sites with statistically sound experimental designs. The resulting plantations are monitored through time by measuring performance-related variables such as tree height and survival. Better adapted populations are expected to grow faster and exhibit higher survival rates. Although a common assumption is that the local provenance is best, this is not always the case. For example, Lu *et al*. showed that white spruce provenances from southern Ontario, Canada, performed better (in terms of height and survival) than local provenances at several northwestern Ontario test sites^1^. There are several potential explanations for the success of non-local provenances, including recent climate change and the migration history of the species^1^.

An important application of provenance data is in the development of seed transfer guidelines^2^. In the past, transfer guidelines have typically aimed to limit seed movements in order to ensure the use of local seed sources at reforestation sites. However, with an evolving climate, directional seed movements (usually poleward or upslope) may be needed to maintain a reasonable match between historical climate at the seed source and future climate at the planting site. Furthermore, optimizing future growth gains obtained by planting non-local provenances must be balanced with possible near-term mortality due to climate/weather-related factors such as winter injury. Since provenance trials gather information on provenance performance across a range of climatic conditions, they are well-suited to provide valuable insights into optimal seed transfer movements under climate change. Unfortunately, tree provenance trials are expensive to establish, maintain, and measure. Further, the long lifespan of trees means that it can take decades to obtain information about mature trees from provenance trials. For these reasons, consolidating existing provenance data into a publicly available repository is an important undertaking.

No current resource provides compiled data from eastern North American tree provenance trials in a single product with associated climate data. Instead, these data are scattered across a variety of sources, including journal articles, conference proceedings, university theses, and government reports. Here we present a data product that consolidates provenance data and associated climate estimates for seven major eastern North American tree species. This effort provides timely data to researchers and forest managers given the recent increased focus on seed transfer under climate change. Prospects for rapid climate change over the coming decades^3^ and management strategies such as assisted migration increase the relevance of historical data^4^. Climate and elevation data are included along with the provenance measurements, in order to, for example, facilitate the calculation of safe climate transfer distances^5^ for each tree species in the database. This article details the data acquisition and data processing methods used to create the databases and describes the structure of the databases and related quality assurance methods. We hope that the databases and information provided in this article will facilitate the use of existing tree provenance data for ongoing seed transfer research and decision making.

## Methods

### Study design & database considerations

We identified seven major tree species that have significant economic and/or ecological value in our geographic area of interest (northeastern United States and eastern Canada): *Picea glauca* (white spruce)*, Picea mariana* (black spruce), *Pinus banksiana* (jack pine), *Larix laricina* (tamarack), *Pinus strobus* (white pine), *Quercus rubra* (red oak), and *Betula alleghaniensis* (yellow birch)). White spruce, black spruce, and jack pine are important commercial conifer species harvested in large volumes across the northern (boreal) portion of our area of interest^6^. Red oak, yellow birch, and white pine are high value hardwood species^7,8^ that have more southern geographic ranges, but have potential for future northward seed transfer. We included data for seed sources from across North America where available, although most originated in the northeastern United States and eastern Canada.

We gathered information for three types of measurement (or response) variables, including tree height, survival, and phenology. Tree height is widely used to indicate provenance growth potential in seed transfer research, but tree survival and phenology data are also important to indicate that a potential seed source can survive and adapt to the conditions at a certain location. Phenology data is of particular interest due to changes in seasonality associated with climate change and the lack of information on how species and populations will respond to these changes^9^. However, this data is not widely reported in tree provenance trials because it is time sensitive and challenging to measure, with multiple visits required to accurately record the phenological stage of the buds^10^.

**Fig. 1:**
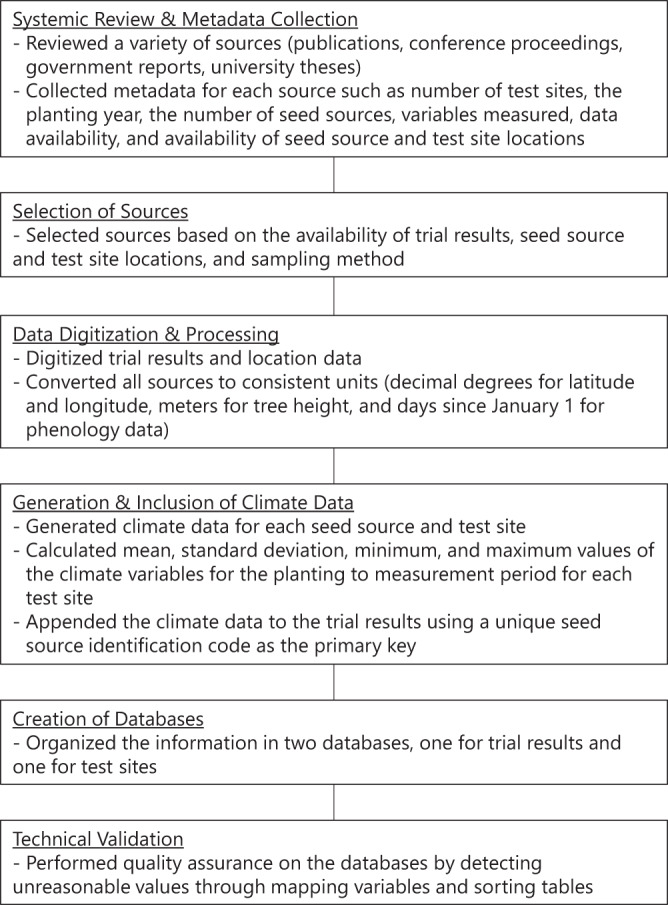
Schematic showing the steps involved in the current study.

In order to identify potential data sources and create a functional database that included both provenance and climate data, we completed six major steps (Fig. 1).

### Systemic review, metadata collection, & selection of sources

We conducted an extensive literature review of sources (publications, conference proceedings, government reports, and university theses) relating to tree provenance trials, for each of the seven tree species of interest. We used keywords such as “provenance trials”, “height growth”, “tree survival”, “common garden study”, and both the common and scientific names of the tree species when searching for sources using resources such as Google Scholar and Reforestation, Nurseries, & Genetic Resources (RNGR). We reviewed a total of 130 sources, recording metadata such as the number of test sites, the year an individual trial was established, the number of provenances included in each trial, provenance mean values of the variables measured in individual trials, tree age at measurement, etc. We also recorded how trees in each trial were measured for each response variable and whether the trial results were reported in-text. If the location information and trial data were reported and it was likely that all trees in the trials were measured, we digitized the information related to the study in a series of tables. Online-only Table 1 provides a summary of sources selected for inclusion in our data product.

### Data processing

All the records in the database were converted to consistent units, such as decimal degrees for latitude and longitude, meters for tree height, and days since January 1 for phenology data. All measurements included in the database represent the mean value for the whole test site, including any replications. For some studies, we assumed this to be the case because no information was provided in the original study about methods used to sample the trees to take measurements. In some cases, there were multiple test sites at one location, representing different environments^11^. In this case, the test sites were entered separately, labeled as “Site 1”, “Site 2”, etc. We also included the test sites separately for different species or studies.

### Climate data associated with provenances and test sites

Estimates for some 65 climate variables (see Online-only Table 2 for more details) were obtained by interrogating existing spatial climate models at each seed source and test site location^12^. Growing season-related variables were calculated using a Bessel interpolation in conjunction with monthly climate variables^13^. The climate estimates included averages for the 1961–1990 period, which provide a reasonable approximation of historical long-term climate values at seed source locations. In addition, climate estimates were generated at each test site for each year over the period spanning plantation establishment and measurement. Specifically, we used a Python program to calculate mean, standard deviation, minimum, and maximum climate values over this period.

Once we generated climate estimates at each test site and seed source location, we used a Python script to append the information (using a unique seed source identification code as the primary key) to the tables containing the provenance trial data. If elevation information had not been reported in the original document, we appended elevation estimates at seed source and test site locations using a digital elevation model (DEM)^14^.

## Data Records

### Data format & storage location

**Fig. 2:**
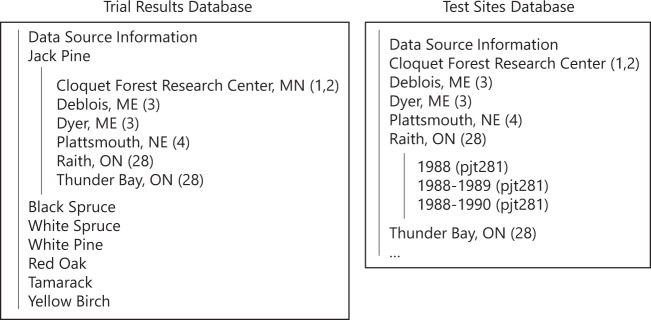
Schematic showing structure of the two databases.

The databases are provided in a series of comma-delimited files (available in both.txt and.csv format). These tables are organized in a series of folders (see Fig. 2). The first database (henceforth the trial results database) contains information on seed sources from 22 provenance studies in eastern North America, including latitude, longitude, elevation, performance measurements (tree height, survival, and phenology data), and 65 long-term climate variables (i.e., averages for the 1961–1990 period). The second database (henceforth the test sites database) contains information on test sites from the same 22 studies, including latitude, longitude, elevation, and 65 climate variables summarized for the period spanning plantation initiation and measurement. All data were recorded in standardized format and units (decimal degrees, meters, °C, etc.). The databases are accessible in an online repository at 10.5063/K072NQ^15^.

Here, we have provided two versions of the trial results database. The first version of the database (trial_results_database) contains tables with consistent columns to facilitate data analysis in environments such as R. If a certain test site did not have a specific measurement, the column was included but contained no data values. This database contains mean height and survival for all trial measurement ages in single columns (i.e., “Mean Height (m)”, “Survival (%)”). To achieve this, we entered multiple records for each seed source. The second version of the database (trial_results_database_primary_key) contains tables with a single row for each seed source to facilitate data analysis with SQL in environments such as Microsoft Access or PostgreSQL. In this case, we included individual columns for each measurement taken at each test site (i.e., “Mean Height Age 10 (m)”). This format means that the seed source identification code can be used as the primary key and can be used to join tables from the same study.

### Data structure & citation

The trial results database is organized by species and contains a table for each test site, identified by both the name of the test site location and the data source number. This database also contains information about the data sources, such as the accuracy of the latitude and longitude of the seed sources (whether it was reported in-text or estimated from the location name) and whether or not phenology data was available. In total, this database contains 75 tables. Table 1 reports an overview of the information contained in this database. Detailed information on the data contained in each table can be found in the database, in the table “Table Overview”.

**Table 1:** Summary of information included in the databases.

Species	Number of Studies	Number of Sites	Number of Sites with Phenology Data	Number of Sites with Survival Data	Number of Sites with Mean Height Data
Jack Pine	4	7	3	2	7
Black Spruce	3	8	0	4	8
White Spruce	4	9	5	8	9
White Pine	4	26	0	2	26
Red Oak	1	7	1	0	7
Tamarack	5	11	0	2	11
Yellow Birch	1	1	0	1	1

The test sites database is organized by test site and includes tables that contain mean, standard deviation, minimum, and maximum climate values for the period between plantation establishment and measurement. If there were multiple measurement years, additional tables were provided for the period between trial establishment and the later measurement years. For example, if a test site was established in 1975 and measured in 1979 and 1980, we included two tables, one for 1975–1979 and one for 1979–1980. In order to avoid tables in the database having the same name, the tables are also named with the unique code of the test site, such as 1988–1990 (pjt281). The purpose of this is to avoid errors when querying the database. The test site database also contains information about the data sources, as well as the test sites (such as whether the elevation data comes from the data source or the DEM). Missing data values were denoted using −9999. In total, this database contains 103 tables. Figure 2 shows a schematic of the folder structure of both databases.

Both databases are organized such that the source of the data is noted alongside the test site name. The full citations of the data sources are found in both databases, in the table named “Guide to Data Sources”. For users of the data, the original source should be cited along with the database.

### Applications

The databases presented here will support seed transfer analyses. The provenance trial data, in combination with the associated climate variables, can be used to calculate critical seed transfer distances – i.e., the distance that a seed source can be moved to improve long term productivity but also minimize the risk of near term mortality^16^. The information provided in the databases may contribute to efforts to develop more robust transfer functions (a function to model climate-performance relationships) by including more test sites and/or seed sources^17^. Furthermore, the datasets can also be used for applications such as mapping trends and finding correlations between climate variables and trial results.

## Technical Validation

**Fig. 3:**
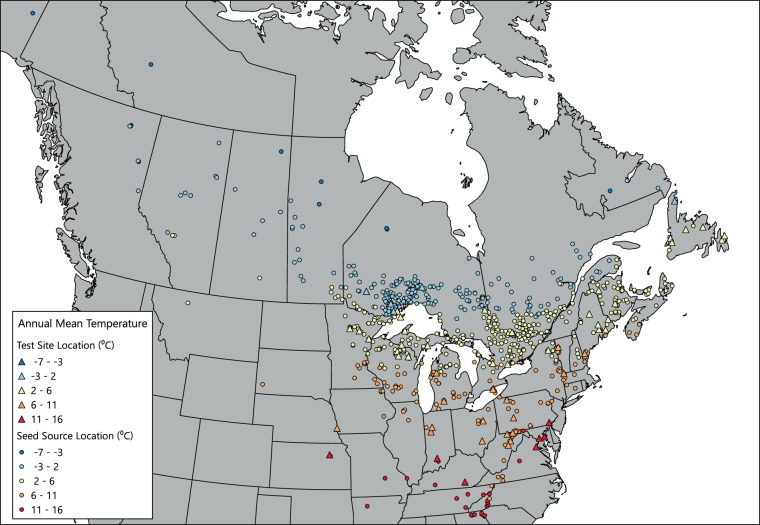
Annual mean (1961–1990) temperature values for seed sources and test sites included in the database.

**Fig. 4:**
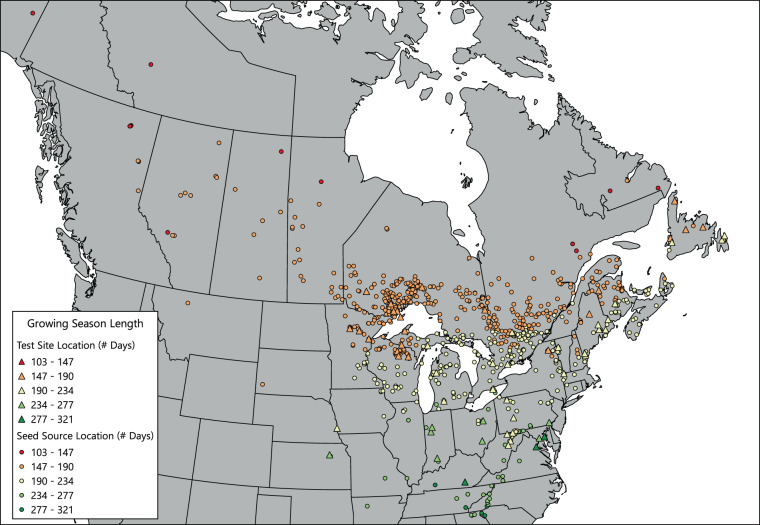
Annual mean (1961–1990) growing season lengths for seed sources and test sites included in the database.

**Fig. 5:**
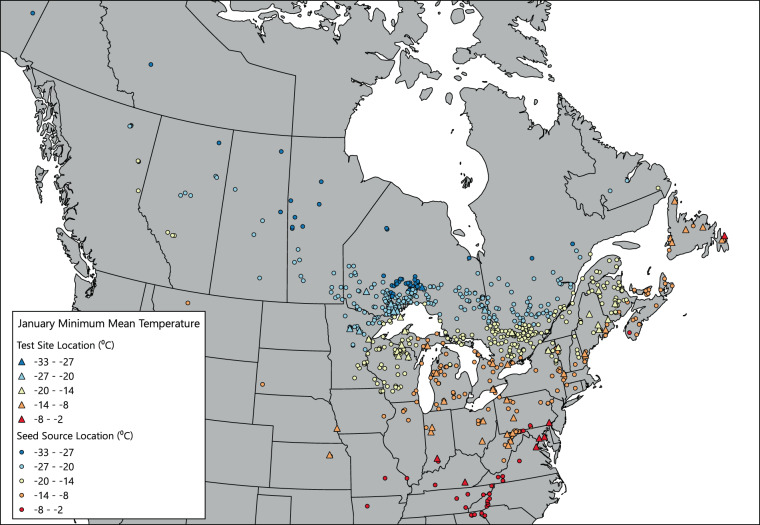
Annual mean (1961–1990) January mean minimum temperature for seed sources and test sites included in the database.

The first method we used for data quality assurance was to examine the data for unreasonable values. For example, tree height mean data for each test site was sorted in ascending and descending order to examine the data for any extreme values. Suspect values were then compared with the source data to check for data entry errors. For quality assurance of the climate data, we mapped the values for mean annual temperature for each provenance and test site in ArcMap (Fig. 3); similar efforts were undertaken for a selection of the other climate variables as well (Figs. 4, 5). Accuracy of the mean, standard deviation, minimum, and maximum calculations was ensured by comparing the results with hand calculations for a selection of data values to ensure that the Python program was working properly.

## Data Availability

We used various Python scripts to process the data for input into the databases. These scripts were used to calculate climate value summaries and convert phenology observations to a uniform reference date (January 1). The scripts are available at https://github.com/clara-risk/tree-provenance-trials.

## Online-only Tables

**Online-only Table 1:** Summary of data sources included in the database.

Data Source	Species	Number of Test Site(s)	# Provenances	Year(s) of Establishment	Year(s) of Measurement
Schantz-Hansen & Jensen (1954);^18^ Shoenike *et al*. (1962)^19^	*Pinus banksiana*	1	12	1942–1943	1950, 1957
Fowler & Heimburger (1969)^20^	*Pinus strobus*	3	12	1963	1965
King & Nienstaedt (1969a);^21^ King & Nienstaedt (1969b)^22^	*Pinus strobus*	5	17	Nursery (Watersmeet, MI) – 1958 Nursery (Rhinelander, WI) – 1960 Test Sites – 1962	Nursery –1958, 1959, 1961 Test Sites – 1966
Garrett *et al*. (1973)^11^	*Pinus strobus*	15	30 total (varies by test site)	1959-1960	1967
Sprackling & Read (1975)^23^	*Pinus banksiana*	1	28	1965	1973
Kriebel *et al*. (1976);^24^ Schlarbaum & Bagley (1976);^25^ Schlarbaum & Bagley (1981);^26^ Kriebel *et al*. (1988)^27^	*Quercus rubra*	7	32 total (varies by test site)	1962–1964	1973–1975
Chech *et al*. (1977)^28^, Chech *et al*. (1982)^29^	*Larix laricina*	2	16	1967	1973, 1981
Clausen (1980)^30^	*Betula alleghaniensis*	1	21	1972	1974, 1975, 1976
Bihun & Carter (1982)^31^	*Picea mariana*	1	99	1976	1981
Hale (1982)^32^	*Picea glauca*	1	28	1965	1979
Hall (1983)^33^	*Larix laricina*	5	2	1967–1976	1982
Park & Fowler (1983)^34^	*Larix laricina*	1	2	1962	1963, 1967, 1971, 1978
Reed *et al*. (1983)^35^	*Larix laricina*	2	43	1967, 1968 (two separate plantations)	1982
Khalil (1984)^36^	*Picea mariana*	3	39 total (varies by test site)	1975	1979, 1980
Carter & Canavera (1985)^37^	*Pinus banksiana*	2	28	1976, 1977	1983
Morgenstern *et al*. (1986)^38^	*Picea mariana*	4	14 (varies by test site)	1973–1977	1985
Genys (1987)^39^	*Pinus strobus*	2	108	1965	1978, 1979
Furnier *et al*. (1991)^40^	*Picea glauca*	1	22	1962	1966, 1976
Farmer *et al*. (1993)^41^	*Larix laricina*	1	6	1986	1987, 1988, 1989, 1990, 1992
van Niejenhuis (1995)^42^	*Pinus banksiana*	2	64	1988	1988, 1989, 1990
Lesser (2005)^10^	*Picea glauca*	6	130	2002	2002, 2003, 2004
Morgenstern *et al*. (2006)^43^	*Picea glauca*	1	25	1963	1975, 2004

**Online-only Table 2:** List of climate variables included in the two databases with summary statistics.

Variable No.	Variable Name	Test Sites Database				Trial Results Database			
		Mean	Standard Deviation	Max	Min	Mean	Standard Deviation	Max	Min
1	Mean Diurnal Range (°C)	11.34	1.51	14.23	7.56	11.68	1.08	15.40	7.90
2	Isothermality 2/7	0.28	0.03	0.37	0.21	0.27	0.03	0.41	0.19
3	Temperature Seasonality (°C)	3.71	0.53	4.86	2.62	4.10	0.59	6.25	2.54
4	Max Temperature Warmest Period (°C)	26.10	2.90	33.25	18.85	24.97	2.20	32.90	14.90
5	Min Temperature Coldest Period (°C)	−14.70	5.27	−5.29	−26.90	−18.42	5.98	−1.60	−32.70
6	Temperature Annual Range (°C)	40.81	4.98	51.33	29.45	43.39	4.97	55.30	28.40
7	Mean Temperature of Wettest Quarter (°C)	10.79	5.78	21.82	0.62	13.74	5.85	22.70	−6.00
8	Mean Temperature Driest Quarter (°C)	1.18	8.08	16.37	−15.23	−7.09	6.87	19.90	−22.50
9	Mean Temperature Warmest Quarter (°C)	18.44	2.79	24.71	12.71	17.17	2.20	24.90	9.30
10	Mean Temperature Coldest Quarter (°C)	−6.88	4.50	1.62	−16.80	−10.90	5.22	5.80	−25.70
11	Annual Precipitation (mm)	949.31	202.10	1372.00	638.67	898.74	213.59	1883.00	278.00
12	Precipitation of Wettest Period (mm)	153.49	26.98	198.47	96.33	104.74	17.00	198.00	56.00
13	Precipitation of Driest Period (mm)	28.40	12.79	50.50	0.00	46.60	21.77	129.00	0.00
14	Precipitation Seasonality (Coefficient of Variation)	49.06	13.03	83.60	33.45	28.90	14.60	84.00	7.00
15	Precipitation of Wettest Quarter (mm)	344.19	58.53	469.83	238.67	294.66	47.40	545.00	151.00
16	Precipitation of Driest Quarter (mm)	141.18	53.75	232.80	42.60	155.96	65.78	422.00	0.00
17	Precipitation of Warmest Quarter (mm)	269.80	42.16	367.27	176.33	279.62	37.92	447.00	133.00
18	Precipitation of Coldest Quarter (mm)	193.78	86.87	369.07	51.60	172.83	80.45	497.00	29.00
19	Start of Growing Season (Julian day)	108.91	19.48	144.27	70.44	115.67	15.20	163.00	44.00
20	End of Growing Season (Julian day)	313.76	14.49	349.73	281.33	304.45	13.21	364.00	250.00
21	Length of Growing Season (# days)	205.85	31.12	280.00	148.67	189.78	27.27	321.00	103.00
22	Total Precipitation for Period 1 (mm)	191.23	72.47	346.41	69.67	167.63	67.65	506.20	31.10
23	Total Precipitation for Period 3 (mm)	597.51	126.17	865.68	349.33	554.62	136.44	1339.40	169.20
24	Gdd Above Base_Temp for Period 3 (Degree days)	1828.65	612.32	3155.00	773.64	1530.62	466.31	3718.00	387.00
25	Annual Mean Temperature (°C)	6.29	3.30	13.04	0.18	3.90	3.33	15.56	−7.17
26	Annual Minimum Temperature (°C)	0.62	3.26	7.81	−6.63	−1.94	3.41	8.91	−13.45
27	Annual Maximum Temperature (°C)	11.96	3.51	19.08	5.91	9.73	3.34	22.20	−0.88
28	Mean Temperature Period 3 (°C)	14.13	1.79	18.36	10.02	13.35	1.33	18.37	8.39
29	Temperature Range for Period 3 (°C)	28.05	2.87	35.49	20.91	26.65	2.15	34.57	16.66
30	Jan Mean Monthly Min Temp (°C)	−13.35	5.19	−4.13	−25.64	−18.38	6.02	−1.61	−32.74
31	Feb Mean Monthly Min Temp (°C)	−12.73	5.06	−3.73	−25.82	−17.00	5.54	−0.31	−29.69
32	Mar Mean Monthly Min Temp (°C)	−6.87	4.43	1.49	−18.81	−10.46	4.87	3.74	−23.60
33	Apr Mean Monthly Min Temp (°C)	−0.32	3.37	6.13	−7.77	−2.42	3.18	8.09	−12.01
34	May Mean Monthly Min Temp (°C)	5.31	3.18	11.78	−0.54	3.95	2.53	12.80	−2.91
35	Jun Mean Monthly Min Temp (°C)	10.43	3.13	16.86	4.52	9.10	2.37	17.18	1.89
36	Jul Mean Monthly Min Temp (°C)	13.41	2.64	19.56	9.18	12.21	2.11	19.79	5.48
37	Aug Mean Monthly Min Temp (°C)	12.54	2.60	19.14	8.50	11.12	2.24	18.94	2.49
38	Sep Mean Monthly Min Temp (°C)	8.55	2.71	15.37	3.89	6.72	2.48	15.67	−3.17
39	Oct Mean Monthly Min Temp (°C)	2.73	2.37	8.54	−3.02	1.36	2.15	8.78	−12.16
40	Nov Mean Monthly Min Temp (°C)	−2.56	3.04	3.62	−10.03	−5.31	3.84	4.19	−25.50
41	Dec Mean Monthly Min Temp (°C)	−9.73	4.74	−0.91	−23.77	−14.20	5.46	0.19	−30.52
42	Jan Mean Monthly Max Temp (°C)	−3.15	4.57	6.42	−13.06	−6.98	5.20	10.75	−23.79
43	Feb Mean Monthly Max Temp (°C)	−1.27	4.17	8.66	−9.45	−4.49	4.52	13.30	−18.65
44	Mar Mean Monthly Max Temp (°C)	4.29	4.22	13.29	−2.93	1.85	3.97	18.25	−10.59
45	Apr Mean Monthly Max Temp (°C)	11.32	4.37	20.12	2.86	9.73	3.40	23.02	−0.33
46	May Mean Monthly Max Temp (°C)	18.29	3.86	25.02	8.34	17.26	2.76	26.80	5.10
47	Jun Mean Monthly Max Temp (°C)	23.42	3.52	29.77	13.81	22.18	2.45	30.23	10.49
48	Jul Mean Monthly Max Temp (°C)	25.89	2.89	33.01	18.64	24.97	2.20	32.94	14.90
49	Aug Mean Monthly Max Temp (°C)	24.60	2.96	31.24	17.86	23.41	2.36	31.88	14.59
50	Sep Mean Monthly Max Temp (°C)	20.37	3.44	26.45	13.36	18.19	2.99	27.98	9.55
51	Oct Mean Monthly Max Temp (°C)	13.94	3.92	21.30	6.71	11.69	3.32	23.00	−2.99
52	Nov Mean Monthly Max Temp (°C)	6.38	3.86	14.24	−0.95	3.29	4.34	17.79	−16.08
53	Dec Mean Monthly Max Temp (°C)	−0.54	4.23	7.95	−10.56	−4.30	4.97	12.47	−21.31
54	Jan Mean Monthly Precipitation (mm)	66.93	34.65	143.43	13.00	56.94	26.86	170.23	8.19
55	Feb Mean Monthly Precipitation (mm)	54.38	26.99	112.89	12.37	48.29	24.84	164.58	8.36
56	Mar Mean Monthly Precipitation (mm)	71.86	28.50	140.21	24.95	59.68	25.67	198.27	7.30
57	Apr Mean Monthly Precipitation (mm)	76.57	24.54	131.73	29.80	63.67	22.59	150.51	7.83
58	May Mean Monthly Precipitation (mm)	85.16	16.46	129.16	53.28	79.79	18.29	163.83	19.69
59	Jun Mean Monthly Precipitation (mm)	87.33	20.51	133.97	38.02	92.02	12.49	142.14	42.80
60	Jul Mean Monthly Precipitation (mm)	92.30	19.27	147.82	54.86	92.92	15.60	155.22	34.08
61	Aug Mean Monthly Precipitation (mm)	92.76	17.58	137.33	58.34	94.66	14.21	149.49	38.69
62	Sep Mean Monthly Precipitation (mm)	89.41	19.77	152.25	43.39	91.94	14.91	144.46	26.48
63	Oct Mean Monthly Precipitation (mm)	79.20	27.06	151.87	25.33	76.63	19.03	161.85	13.01
64	Nov Mean Monthly Precipitation (mm)	78.44	29.28	150.70	18.50	74.15	27.74	182.99	10.67
65	Dec Mean Monthly Precipitation (mm)	75.00	33.00	152.36	21.51	68.04	31.22	192.21	10.05

Variables #1-#18; #30-#65 are derived from Xu & Hutchinson 2010^44^, variables #19-#29 are derived from McKenney *et al*. 2011^12^ and Mackey *et al*. 1996^13^. The summary statistics include the mean, standard deviation, maximum, and minimum values from the test sites database for the time period when each site was active and from the trial results database for the 1961–1990 period for each seed source location with climate data available.
